# MiR-30a upregulates BCL2A1, IER3 and cyclin D2 expression by targeting FOXL2

**DOI:** 10.3892/ol.2014.2723

**Published:** 2014-11-20

**Authors:** TAIREN WANG, FEI LI, SHENGJIAN TANG

**Affiliations:** Institute of Plastic Surgery, Weifang Medical College, Weifang, Shandong 261041, P.R. China

**Keywords:** FOXL2, microRNA, miR-30a, ovarian granulosa cell tumor

## Abstract

FOXL2 is a transcription factor that is essential for ovarian development. Somatic mutations of FOXL2 are associated with ovarian granulosa cell tumorigenesis. In the present study, the expression of FOXL2 was suppressed by microRNAs using the Ago2 knockdown method in COV434 cells. Online bioinformatics tools were utilized to predict that FOXL2 expression may be repressed by miR-30 family members, and dual luciferase assay and western blotting were performed to demonstrate that FOXL2 is a target gene of miR-30a, which is relatively abundant in COV434 cells. Furthermore, miR-30a overexpression upregulates BCL2A1, IER3 and cyclin D2 expression by inhibiting FOXL2. miR-30a is known to function as a tumor suppressor in breast cancer, small cell lung cancer and colorectal carcinoma; however, the present study revealed an opposing function of miR-30a as an oncogene.

## Introduction

FOXL2, a Forkhead box family transcription factor initially described in *Drosophila*, is predominantly expressed in periocular, ovarian and pituitary cells. FOXL2 was first cloned and localized by Crisponi *et al* (1) and is mutated in blepharophimosis ptosis epicanthus inversus syndrome (BPES), a genetic disorder characterized by eyelid malformations. More than two-thirds of BPES patients carry intragenic FOXL2 mutations and one-third of mutations in the FOXL2 coding region are expansions of the polyalanine tract, from 14 to 24 residues (2,3). Furthermore, heterozygous mutations in FOXL2 result in premature ovarian failure and infertility in females (4).

FOXL2 functions as a transcriptional repressor and requires sumoylation and phosphorylation for its activity (5,6). Somatic mutations of FOXL2 have been reported to reduce its activity and may be associated with enhanced cancer cell proliferation, accelerated cell cycle progression and reduced sensitivity to apoptosis (7). In addition, genes that are differentially expressed in ovarian granulosa cell tumors (GCTs) are significantly enriched for known FOXL2 target genes, consistent with the prevalence of FOXL2 somatic mutations in these tumors (8).

MicroRNAs (miRNAs) are short non-coding RNAs which modulate gene expression by binding to complementary areas in the 3′-untranslated region of protein-coding gene mRNA. miRNAs are important in maintaining normal physiological conditions in humans, and abnormal miRNA expression has been associated with numerous human diseases, including psychiatric disorders and malignant cancer (9–11). Bioinformatics research has indicated that all of the miRNAs may target >60% of mammalian protein-coding genes (12).

The present study used bioinformatic tools to predict miRNAs that may directly target FOXL2. Among them, miR-30 family members are associated with human ovarian carcinogenesis (13). Subsequently, dual luciferase assays and western blotting identified FOXL2 as the target gene of miR-30a. Furthermore, miR-30a upregulated BCL2A1, IER3 and cyclin D2 expression by repressing FOXL2 expression.

## Materials and methods

### Small interfering (si)RNA knockdown

siRNAs against Ago2, miR-30a mimic and miR-30a inhibitor were purchased from RiboBio Co., Ltd. (Guangzhou, China) and transfected into COV434 cells at a concentration of 200 nM.

### Cell culture

The human granulosa COV43 cells (Shanghai Insitutes for Biological Sciences, Chinese Academy of Sciences, Shanghai, China) were cultured in Dulbecco’s modified Eagle medium supplemented with 10% fetal bovine serum (HyClone Laboratories, Inc., Logan, UT, USA), 100 IU/ml penicillin and 10 mg/ml streptomycin. All cells were maintained at 37°C in an atmosphere of 5% CO_2_.

### RNA extraction and detection of miR-30 family member expression

Reverse transcription-quantitative polymerase chain reaction (RT-qPCR) was performed to determine the relative expression level of specific miR-30 family members (miR-30a/b/c/d/e). Total RNA was extracted from COV434 cells using TRIzol reagent (Invitrogen Life Technologies, Carlsbad, CA, USA), according to the manufacturer’s instructions. The expression levels of the various miRNAs were detected using TaqMan^®^ RT-qPCR miRNA assays. Single-stranded complementary DNA was synthesized using a TaqMan MicroRNA Reverse Transcription kit, and amplified using TaqMan Universal PCR Master mix and miRNA-specific TaqMan MGB probes (all TaqMan products were purchased from Applied Biosystems Life Technologies, Foster City, CA, USA). U6 small nuclear RNA was used for data normalization. Each sample was measured in triplicate and the experiment was repeated a minimum of three times to ensure miRNA detection.

### Western blotting

Protein extracts were boiled in SDS/β-mercaptoethanol (2:1; w/v) sample buffer, and 30 μg samples were loaded into each lane and separated by electrophoresis on 8% polyacrylamide gels. The separated proteins were electrophoretically transferred onto polyvinylidene fluoride membranes (GE Healthcare Life Sciences, Chalfont, UK), which were incubated with goat anti-human FOXL2 polyclonal antibody (1:1,000; cat. no., ab5096; Abcam, Cambridge, MA, USA) and mouse anti-human AGO2 monoclonal antibody (1:1,000; cat. no., ab57113; Abcam) or mouse anti-human β-actin monoclonal antibody (1:3,000; cat. no., sc-69879; Santa Cruz Biotechnology Inc., Santa Cruz, CA, USA) for 1 h at 37°C. The specific protein antibody complex was detected using horseradish peroxidase-conjugated rabbit anti-goat and rabbit anti-mouse polyclonal IgG secondary antibody (1:5,000; Santa Cruz Biotechnology, Inc.) and visualized using an enhanced chemiluminescence kit (Pierce Manufacturing Inc., Appleton, WI, USA). The β-actin signal was used as the loading control.

### Dual luciferase assay

The full length FOXL2 3′-UTR (1,129 bp) was cloned into a pGL3 control vector (Promega Corporation, Madison, WI, USA), downstream of the firefly luciferase coding region, to generate a luciferase reporter vector. For luciferase reporter assays, COV434 cells were seeded in 48-well plates. An miR-30a mimic or miR-30a inhibitor were co-transfected with luciferase reporter vectors (Promega Corporation) using Lipofectamine 2000 (Invitrogen Life Technologies). Cells were harvested after two days and assayed using the Dual-Luciferase^®^ Reporter Assay system (Promega Corporation) to determine the relative luciferase activity (LUC) of the COV434 cells. pRL-TK containing Renilla luciferase was co-transfected with the 3′-UTR of FOXL2 for data normalization. Each treatment was performed in triplicate in three independent experiments and LUC was expressed as firefly LUC/Renilla LUC.

### Statistical analysis

Data were analyzed using SPSS statistical software (version 16; SPSS, Inc., Chicago, IL, USA). Independent analysis between the two groups was performed using a t-test. P<0.05 was considered to indicate a statistically significant difference.

## Results

### FOXL2 expression is regulated by miRNAs

**Figure 1:**
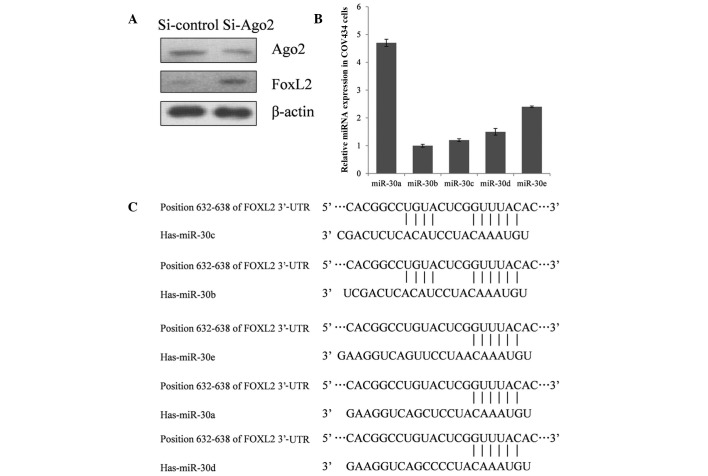
FOXL2 expression was significantly repressed by miRNAs in COV434 cells. (A) FOXL2 expression was upregulated when AGO2 was knocked down; therefore, FOXL2 expression was repressed by endogenous microRNAs. (B) Reverse transcription-quantitative polymerase chain reaction of miR-30a/b/c/d/e expression in COV434 cells revealed that miR-30a is relatively abundant compared with other miR-30 family members. (C) The predicted interactions between miR-30a/b/c/d/e and FOXL2 3′-UTR mRNA. miR/miRNA, microRNA; 3′-UTR; 3′-untranslated region.

To explore whether the expression of FOXL2 is regulated by miRNAs, Ago2, a key component of the RNA-induced silencing complex, was knocked down in COV434 cells. This knockdown demonstrated that inactivation of the miRNA system results in upregulation of FOXL2 expression (Fig. 1A), indicating that miRNAs are involved in the negative control of FOXL2 expression.

### miR-30a represses FOXL2 expression by binding to 3′-UTR

**Figure 2:**
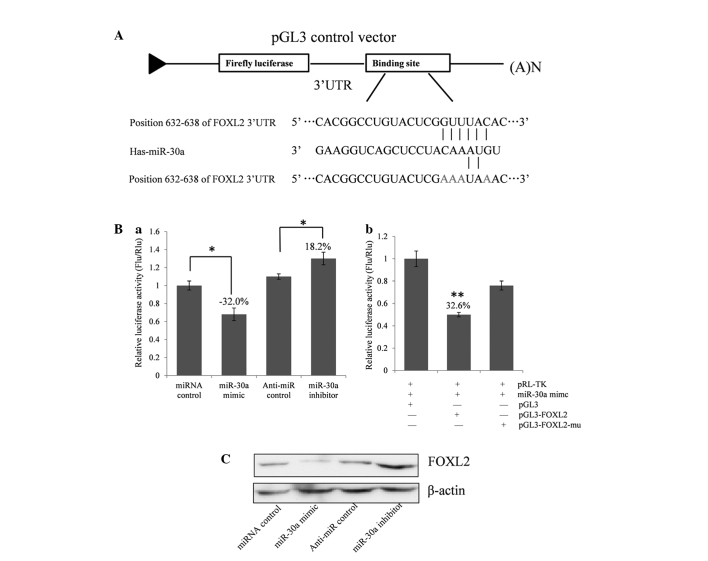
FOXL2 is a target gene of miR-30a. (A) Schematic diagram for constructing the predicted miR-30a binding site into the pGL3 control vector. (B) Histograms indicating the target gene of miR-30a. (a) A dual luciferase assay was conducted of COV434 cells co-transfected with the pGL3-FOXL2 and an miRNA control, miR-30a mimic, anti-miR control or miR-30a inhibitor. pRL-TK containing Renilla luciferase was co-transfected with the FOXL2 3′-UTR data normalization. ^*^P<0.05. (b) Mutational analysis of the miR-30a binding site. Luciferase activity was significantly decreased in COV434 cells co-transfected with miR-30a mimics and pGL3-FOXL2 compared with pGL3-FOXL2-Mu (a four-nucleotide mutation of the miR-30a binding site in the FOXL2 3′-UTR) or pGL3. ^**^P<0.01. (C) Western blot analysis was used to detect changes in FOXL2 protein expression levels in the miR-30a mimic or inhibitor-treated COV434 cells. miR/miRNA, microRNA; 3′-UTR; 3′-untranslated region.

TargetScan Release 6.2 (http://www.targetscan.org/), an online tool for predicting the interaction between miRNAs and genes, was used to probe miRNAs which may suppress FOXL2 expression. Of the candidate miRNAs, miR-30 family members were reported to be associated with various types of human cancer, and the expression of miR-30a was high in COV434 cells, compared with the other miRNAs evaluated (Fig. 1B). Thus, miR-30a was selected for further investigation of its role in the repression of FOXL2 expression. As demonstrated in Fig. 2A, a the full length 3′-UTR of FOXL2 was cloned into the pGL3 control plasmid, downstream of the firefly luciferase coding region, and a dual luciferase assay was conducted. COV434 cells were co-transfected with pGL3-FOXL2 and miR-30a mimics or inhibitors (Fig. 2Ba). The present study identified that luciferase activity was significantly suppressed by the miRNA control compared with the miR-30a mimic (~32.0%; P<0.05). Furthermore, luciferase activity was significantly upregulated by the miR-30a inhibitor compared with the anti-miR control (~18.2%; P<0.05). These results indicate that miR-30a targets the 3′-UTR of FOXL2, resulting in altered translation of the firefly luciferase gene.

A seed sequence mutation clone was used to clarify the location of the miR-30a binding site (Fig. 2A). A four-nucleotide mutation in the putative miR-30a binding region of the FOXL2 3′-UTR (termed, pGL3-FOXL2-Mu) and an empty pGL3 vector were used as the controls. The histogram in Fig. 2Bb demonstrates that the relative luciferase activity was reduced by ~32.6% in cells co-transfected with the miR-30a mimic and pGL3-FOXL2 compared with the miR-30a mimic and pGL3-FOXL2-Mu (P<0.01). These data indicate that miR-30a may suppress FOXL2 gene expression through binding to the seed sequence at the 3′-UTR of FOXL2, and that FOXL2 may be a direct target of miR-30a.

### miR-30a regulates endogenous FOXL2 expression in COV434 cells

Although FOXL2 was identified as a target gene for miR-30a, it was unknown whether miR-30a could regulate endogenous FOXL2 expression. COV434 cells were transfected with miR-30a mimics or inhibitors to investigate whether the dysregulation of miR-30a expression affected endogenous FOXL2 expression. Compared with the corresponding control, the level of FOXL2 protein was significantly suppressed by miR-30a mimics and upregulated by miR-30a inhibitors (Fig. 2C).

### miR-30a promotes BCL2A1, IER3 and cyclin D2 gene expression by suppressing FOXL2

**Figure 3:**
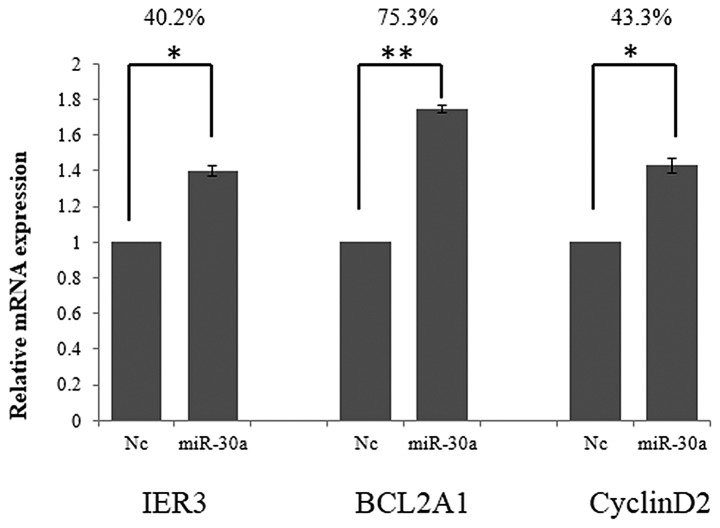
Reverse transcription-quantitative polymerase chain reaction was used to detect that miR-30a overexpression upregulates relative BCL2A1, IER3 and cyclin D2 mRNA expression by 40.2, 75.3 and 43.3%. ^*^P<0.05 and ^**^P<0.01. miR/miRNA, microRNA; Nc, normal control.

The consequences of miR-30a knockdown and overexpression indicate that miR-30a regulates cell function by regulating FOXL2. To further investigate this, the effects of miR-30a treatment on IER3, BCL2A1 and cyclin D2 were investigated. IER3, BCL2A1 and cyclin D2 mRNA expression were detected using RT-qPCR, 48 h after transfection with miR-30a. As demonstrated in Fig. 3, the relative mRNA expression of IER3, BCL2A1 and cyclin D2 was significantly increased by 40.2, 75.3 and 43.3%, respectively, following miR-30a overexpression.

## Discussion

FOXL2 is involved in craniofacial and female genital system development, and FOXL2 mutations can result in the development of BPES, ovary failure and GCTs (2,14,15). Studies have indicated that FOXL2 is significantly downregulated in the COV434 GCT cell line, despite no alterations to the genomic DNA (16,17). Therefore, the present study aimed to investigate the negative control system of FOXL2 expression. Since miRNAs regulate a large proportion of protein-coding genes, Ago2 was initially knocked down to evaluate the effect of miRNAs on FOXL2 expression. This Ago2 knockdown resulted in a significantly upregulation of FOXL2 expression. Following prediction using bioinformatics tools and clarification by dual luciferase assay and western blotting, miR-30a was identified to repress FOXL2 in COV434 cells. Furthermore, this repression function was reflected in the upregulation of genes regulated by FOXL2.

As a transcriptional repressor, FOXL2 suppresses BCL2A1, IER3 and cyclin D2 gene expression in granulosa cells (18–20). BCL2A1 and IER3 are apoptosis inhibitors, whereas cyclin D2 is a cell cycle-associated gene which regulates G1/S transition. High expression levels of these genes have been observed in ovarian and testicular tumors.

miRNA is a type of post-transcriptional suppressor and a single miRNA may regulate hundreds of protein-coding genes. In particular, miR-30a acts as a tumor suppressor in breast cancer, non-small cell lung cancer and colorectal carcinoma (21–23). However, the present study identified that miR-30a also acts as an oncogene by repressing FOXL2 expression in GCTs. In conclusion, further investigation is required to expand on the present research and evaluate the function of miR-30a in granulosa cell tumors and other types of cancer.

## References

[1] Crisponi L, Uda M, Deiana M (2004). FOXL2 inactivation by a translocation 171 kb away: analysis of 500 kb of chromosome 3 for candidate long-range regulatory sequences. Genomics.

[2] De Baere E, Beysen D, Oley C (2003). FOXL2 and BPES: mutational hotspots, phenotypic variability, and revision of the genotype-phenotype correlation. Am J Hum Genet.

[3] De Baere E, Dixon MJ, Small KW (2001). Spectrum of FOXL2 gene mutations in blepharophimosis-ptosis-epicanthus inversus (BPES) families demonstrates a genotype - phenotype correlation. Hum Mol Genet.

[4] Zlotogora J, Sagi M, Cohen T (1983). The blepharophimosis, ptosis, and epicanthus inversus syndrome: delineation of two types. Am J Hum Genet.

[5] Benayoun BA, Batista F, Auer J (2009). Positive and negative feedback regulates the transcription factor FOXL2 in response to cell stress: evidence for a regulatory imbalance induced by disease-causing mutations. Hum Mol Genet.

[6] Pisarska MD, Kuo FT, Bentsi-Barnes IK, Khan S, Barlow GM (2010). LATS1 phosphorylates forkhead L2 and regulates its transcriptional activity. Am J Physiol Endocrinol Metab.

[7] Pisarska MD, Barlow G, Kuo FT (2011). Minireview: roles of the forkhead transcription factor FOXL2 in granulosa cell biology and pathology. Endocrinology.

[8] Benayoun BA, Anttonen M, L’Hôte D (2013). Adult ovarian granulosa cell tumor transcriptomics: prevalence of FOXL2 target genes misregulation gives insights into the pathogenic mechanism of the p. Cys134Trp somatic mutation. Oncogene.

[9] Maes OC, Chertkow HM, Wang E, Schipper HM (2009). MicroRNA: Implications for Alzheimer disease and other human CNS disorders. Curr Genomics.

[10] Xu J, Li Y, Wang F (2013). Suppressed miR-424 expression via upregulation of target gene Chk1 contributes to the progression of cervical cancer. Oncogene.

[11] Farazi TA, Hoell JI, Morozov P, Tuschl T (2013). MicroRNAs in human cancer. Adv Exp Med Biol.

[12] Friedman RC, Farh KK, Burge CB, Bartel DP (2009). Most mammalian mRNAs are conserved targets of microRNAs. Genome Res.

[13] Lee H, Park CS, Deftereos G (2012). MicroRNA expression in ovarian carcinoma and its correlation with clinicopathological features. World J Surg Oncol.

[14] Corrêa FJ, Tavares AB, Pereira RW, Abrão MS (2010). A new FOXL2 gene mutation in a woman with premature ovarian failure and sporadic blepharophimosis-ptosis-epicanthus inversus syndrome. Fertil Steril.

[15] Kim JH, Yoon S, Park M (2011). Differential apoptotic activities of wild-type FOXL2 and the adult-type granulosa cell tumor-associated mutant FOXL2 (C134W). Oncogene.

[16] Kalfa N, Fellous M, Boizet-Bonhoure B (2008). Aberrant expression of ovary determining gene FOXL2 in the testis and juvenile granulosa cell tumor in children. J Urol.

[17] Kalfa N, Philibert P, Patte C (2007). Extinction of FOXL2 expression in aggressive ovarian granulosa cell tumors in children. Fertil Steril.

[18] D’Sa-Eipper C, Chinnadurai G (1998). Functional dissection of Bfl-1, a Bcl-2 homolog: anti-apoptosis, oncogene-cooperation and cell proliferation activities. Oncogene.

[19] Wu MX, Ao Z, Prasad KV, Wu R, Schlossman SF (1998). IEX-1L, an apoptosis inhibitor involved in NF-kappaB-mediated cell survival. Science.

[20] Bentsi-Barnes IK, Kuo FT, Barlow GM, Pisarska MD (2010). Human forkhead L2 represses key genes in granulosa cell differentiation including aromatase, P450scc, and cyclin D2. Fertil Steril.

[21] Zhang N, Wang X, Huo Q (2014). MicroRNA-30a suppresses breast tumor growth and metastasis by targeting metadherin. Oncogene.

[22] Jiang BY, Zhang XC, Su J (2013). BCL11A overexpression predicts survival and relapse in non-small cell lung cancer and is modulated by microRNA-30a and gene amplification. Mol Cancer.

[23] Zhong M, Bian Z, Wu Z (2013). miR-30a suppresses cell migration and invasion through downregulation of PIK3CD in colorectal carcinoma. Cell Physiol Biochem.

